# Hypnotic Discontinuation Using a Blinded (Masked) Tapering Approach: A Case Series

**DOI:** 10.3389/fpsyt.2019.00717

**Published:** 2019-10-24

**Authors:** Constance H. Fung, Jennifer L. Martin, Cathy Alessi, Joseph M. Dzierzewski, Ian A. Cook, Alison Moore, Austin Grinberg, Michelle Zeidler, Lara Kierlin

**Affiliations:** ^1^Geriatric Research, Education and Clinical Center, VA Greater Los Angeles Healthcare System, North Hills, CA, United States; ^2^Department of Medicine, David Geffen School of Medicine at the University of California, Los Angeles (UCLA), Los Angeles, CA, United States; ^3^Department of Psychology, Virginia Commonwealth University, Richmond, VA, United States; ^4^Semel Institute for Neuroscience and Human Behavior at UCLA, Los Angeles, CA, United States; ^5^Los Angeles TMS Institute, Los Angeles, CA, United States; ^6^Department of Medicine, University of California, San Diego School of Medicine, La Jolla, CA, United States; ^7^Northwest Sleep and Behavior, Lake Oswego, OR, United States

**Keywords:** benzodiazepine discontinuation, cognitive behavioral therapy for insomnia, placebo effect, nocebo effect, medication taper

## Abstract

Chronic use of hypnotic medications such as benzodiazepines is associated with adverse consequences including increased risk of falls. Efforts to help patients discontinue these medications have had varying levels of success. We developed a blinded (masked) tapering protocol to help patients taper off hypnotics. In this blinded protocol, patients consented to a drug taper but agreed to forego knowledge about the specific tapering schedule or the actual dose each night until the end of the taper. Blinded tapering aims to reduce negative expectancies for withdrawal effects that may impair a patient’s successful discontinuation of hypnotics. In preparation for a randomized trial, we tested the feasibility of adding a blinded tapering component to current best evidence practice [supervised hypnotic taper combined with cognitive behavioral therapy for insomnia (CBTI)] in 5 adult patients recruited from an outpatient mental health practice in Oregon. A compounding pharmacy prepared the blinded capsules for each patient. During the gradual blinded taper, each participant completed CBTI. Measures collected included feasibility/process (e.g., recruitment barriers), hypnotic use, the Dysfunctional Beliefs and Attitudes about Sleep Scale, Insomnia Severity Index, Epworth Sleepiness Scale, and Patient Health Questionnaire-9 (depressive symptoms). The intervention was feasible, and participants reported high satisfaction with the protocol and willingness to follow the same treatment again. All five participants successfully discontinued their hypnotic medication use post-treatment. Dysfunctional beliefs/attitudes about sleep and insomnia severity improved. Blinded tapering is a promising new method for improving hypnotic discontinuation among patients treated with a combination of hypnotic tapering plus CBTI.

## Introduction

Clinical guidelines recommend non-pharmacological therapy such as cognitive behavioural therapy for insomnia (CBTI) as first-line treatment of chronic insomnia disorder in all adults (1). Pharmacological therapy for insomnia, however, is common (2). Chronic use of hypnotic medications such as benzodiazepines is associated with adverse consequences such as increased risk of falls (3, 4). Discontinuation of hypnotics such as benzodiazepines and benzodiazepine receptor agonists is recommended, particularly in older adults (5) and patients who have not tried non-pharmacological therapy. Published studies have employed a variety of discontinuation strategies, including supervised dose reduction, and have established the safety of supervised reduction approaches (6). The efficacy of supervised dose reduction alone, however, is modest, and therefore, approaches that combine supervised dose reduction with psychological interventions such as CBTI have been recommended. Unfortunately, studies examining the combination of these two treatment methods have yielded mixed results (7).

One factor that may interfere with dose reduction of a hypnotic may be discomfort related to withdrawal symptoms from pharmacological effects. However, pharmacological effects likely do not entirely explain the symptoms that patients report when tapering a hypnotic medication or similar agent (8). Negative expectations about stopping an agent have the potential to produce withdrawal symptoms (9). A strategy to overcome these expectations is blinded (or masked) tapering of a drug; this entails gradually tapering the drug without providing the patient with explicit knowledge of the taper schedule.

This study was designed to test the feasibility of adding a blinded tapering component to an existing protocol that combines supervised tapering of hypnotics with CBTI. Our primary aim was to gather feasibility/process information needed to design a randomized trial testing the benefits of blinded versus standard tapering. Our secondary aim was to measure differences in hypnotic use and attitudes about sleep and hypnotics from baseline to post-treatment. Our *a priori* hypotheses were that hypnotic use would decrease from baseline to post-treatment and that attitudes and beliefs about sleep and hypnotics would improve from baseline to post-treatment.

## Patients and Methods

### Overview

Our study design was a case series intended to assess the feasibility of adding a blinded tapering component to a protocol involving a supervised gradual hypnotic taper plus CBTI. Between February 2014 and October 2014, patients were recruited from a community outpatient mental health practice. Adult patients with chronic hypnotic use who were referred for treatment of chronic insomnia were provided a study information sheet at their initial appointment and given the opportunity to ask questions about the study. Interested patients were invited to give full informed consent to the study protocol that included CBTI concurrent with blinded tapering of their current sleep medication. Patients were fully informed that during the study they would not know the amount of hypnotic they would be taking each night, but would be informed of the tapering schedule at the end of the study. Follow-up was limited to a single post-treatment assessment because the purpose of the study was to assess feasibility of the intervention and to test blinding procedures. No financial incentives were provided to participants. Participants were responsible for usual treatment costs (e.g., co-payments) associated with usual care, including office visits and medications. All research activities were approved by the Providence Health and Services Institutional Review Board.

### Eligibility Criteria

Patients who expressed interest in participating were screened for eligibility criteria through a clinical assessment interview with the study principal investigator. The eligibility criteria were: age ≥18 years old, current insomnia disorder according to insomnia diagnostic criteria of the International Classification of Sleep Disorders, 2^nd^ edition (10) (which were the criteria in place at the time), current use of benzodiazepine or non-benzodiazepine benzodiazepine receptor agonist or trazodone for sleep on more than 50% of nights for at least 3 months, and availability to attend 6 weeks of in-person CBTI. Patients were excluded if they: had a history of sleep apnea of moderate or severe severity (apnea–hypopnea index ≥ 15) or periodic limb movements during sleep [periodic limb movement with arousal ≥ 15 (10)] based on a prior sleep study, were currently participating in psychotherapy with a provider other than the study therapist, were using psychotropic drugs other than the study medications or over the counter sleep agents, had severe mental illness including major depressive disorder with active suicidal ideation, bipolar disorder, current psychosis, or current alcohol/substance abuse, or allergy or intolerance to both lactose and corn (which were in the inert fillers used by the compounding pharmacy to prepared the study medication capsules).

### Measures

Demographic measures included age, gender, living arrangement, level of education, and employment status. The duration of hypnotic use and number of prior attempts to discontinue sedative-hypnotic medications was also collected. Expectations and credibility of the study program (11) were collected at baseline and post-treatment (immediately after the treatment was completed). Sleep onset latency, wake after sleep onset, time in bed, and total sleep time were collected daily in a sleep diary during intervention. Participants were also asked to record on their sleep diary the dose they believed they were taking each night (“guessed dose”). At post-treatment, satisfaction was assessed with the following items: “I was satisfied with the medication reduction method” and “I would be willing to follow the same treatment again if necessary” [with response options ranging from “Strongly agree” (0) to “Strongly disagree” (3)].

Several structured questionnaires were performed at baseline and post-treatment. The Epworth Sleepiness Scale measured hypersomnolence (8 items; score range 0–24; higher scores suggest more daytime sleepiness) (12). The Insomnia Severity Index measured insomnia severity (13). The Dysfunctional Beliefs and Attitudes Scale-16 (DBAS-16; 16 items; score range 0–160; higher scores suggest more dysfunctional beliefs) (14) was also administered, with a mean score ≥ 4 indicating unrealistic expectations about sleep (15). The Patient Health Questionnaire-9 was collected to measure depressed mood (9 items; score range 0–27; higher scores are associated with worse mood) (16). During the treatment, the Clinical Institute Withdrawal Assessment Scale-Benzodiazepine (17) was administered to test for symptoms of withdrawal (20 items; score range 0-80; higher scores are associated with more withdrawal symptoms).

### Intervention Description

**Table 1** summarizes the intervention, which was received by all participants. Participants were informed they would receive a supervised hypnotic tapering protocol, with the goal of eliminating hypnotic use by the sixth week of treatment (18–20). Participants were not informed of either their particular tapering protocol or the actual dose of hypnotic they would receive each night. Each participant’s tapering schedule was individualized according to the type of hypnotic, baseline dose, and frequency of use. Several principles were employed during intervention development, including: 1) assisting participant in setting goals, 2) (if applicable) stabilization of hypnotic use to one hypnotic agent if more than one hypnotic in use (in this case series, no participants were on more than one type of hypnotic at baseline), 3) (if applicable) conversion of hypnotic use from as-needed to a schedule of nightly capsule use, and 4) reduction of the initial dose by roughly 25% every 1 to 2 weeks until the lowest available dose of active drug was reached. The actual capsules were provided by a compounding pharmacy, were not marked with dose information, and contained a step-wise decrease in the dose of active hypnotic medication plus a step-wise increase in the amount of inactive filler (either lactose or corn starch, depending upon whether the patient reported a history of allergy or intolerance to either substance). In addition, the protocol included the introduction of an increasing number of reduced drug nights each week through blinded tapering (3). The actual dose reductions in the individualized tapering protocol schedules varied based upon each patient’s readiness to discontinue the hypnotic (reported at baseline) and adjusted (if necessary) based on findings from weekly monitoring. At the end of treatment, all participants would expect that the dose in the final capsule was zero milligrams. For participants taking a benzodiazepine or benzodiazepine receptor agonist, we monitored for the presence or absence of withdrawal symptoms (based on Clinical Institute Withdrawal Assessment Scale-Benzodiazepine (17) for participants taking a benzodiazepine or benzodiazepine receptor agonist). The capsules that were prepared by a local licensed compounding pharmacy were given to participants in a blister pack to ensure the correct capsule ingested on the correct date and that no information on dosage was revealed to the participant.

**Table 1:** Cognitive behavioral therapy for insomnia and supervised hypnotic tapering protocol.

Week	CBTI (in-person on Day 1 of each week)	Medication
1	Overview of methods involved, a review of mechanisms that control sleep, and an introduction to stimulus control and sleep restriction as well as overview of sleep hygiene. Instructions on completion of the sleep diary	During each day of week 1, the probability of taking a capsule that contains full strength hypnotic is 50% and the probability of taking a capsule that contains ¾ strength was 50%*.
2	Sleep diary data reviewed. Adjustments to sleep plan made based upon calculated sleep efficiency (i.e., time asleep over time in bed [TIB] X 100). Per standardized CBTI program, participant time in bed (TIB) was adjusted to achieve a goal sleep efficiency of greater than 85%. CBTI troubleshooting of difficulties with the program, as well as addressing barriers to adherence (e.g., anxiety, problems with sleep restriction).	During each day of week 2, the probability of taking a capsule that contains ¾ strength hypnotic is 50% and the probability of taking a capsule that contains ½ strength was 50%.
3	Sleep diary data were reviewed and adjustments made to TIB to reflect either desire for more sleep (if 85% sleep efficiency has been met) or reduction of TIB (if sleep efficiency of 85% has not been achieved). Also involves discussion of relaxation techniques as well as paradoxical intention (i.e., an approach that involves instruction to try to stay awake)	During each day of week 3, the probability of taking a capsule that contains ½ strength hypnotic is 50% and the probability of taking a capsule that contains ¼ strength was 50%.
4	Sleep diary data were reviewed and adjustments made to TIB to reflect either desire for more sleep or reduction in TIB as above. Any new/ongoing problems with adherence were also reviewed and addressed.	During each day of week 4, the probability of taking a capsule that contains 1/4 strength hypnotic is 50% and the probability of taking a capsule that contains 0%strength was 50%.
5	Sleep diary data were reviewed and adjustments made to TIB to reflect either desire for more sleep or reduction in TIB as described above. Any new/ongoing problems with adherence were also reviewed and addressed.	During each day of week 5, the probability of taking a capsule that contains 1/4 strength hypnotic is 50% and the probability of taking a capsule that contains 0 strength was 50%.
6	Sleep diary data reviewed. Participants presented with instructions for contingency planning CBTI if sleep is derailed in the future. Participants had the opportunity to ask questions and were made aware that they may return on an as-needed basis.	During each day of week 6, the probability of taking a capsule that contains 0 strength was 100%. Participant informed of the protocol they received after completing the final week

CBTI, cognitive behavioral therapy for insomnia. *Within a single week, the dose may vary from day-to-day (e.g., the dose could be ¾ strength on day 1 and full-strength on day 2 and ¾ strength on day 3).

Participants received six sessions of CBTI delivered by a licensed psychiatrist/sleep medicine physician with specific specialty training in CBTI.

## Results

### Sample Characteristics

Six patients were provided detailed information about the study. Of these six patients, one declined due to perceived “psychological dependence” on the hypnotic. Five patients (4 female; 1 male) were consented and enrolled. **Table 2** summarizes the cases. The mean age was 55.9 years (SD 19.6; range 49–72 years). Four patients lived with their spouse. One patient lived with his/her spouse and other family. The mean years of formal education was 17.2 years (SD 4.1; range 14 to 22 years). No participants were currently employed at the time of the study.

**Table 2:** Description of cases.

Case number	Age	Medication	Number of years of hypnotic use	Number of prior attempts to discontinue hypnotic use
1	64	trazodone 25 mg	10	2
2	49	lorazepam 1 mg	30	10
3	24	zolpidem extended release 6.25 mg	8	1
4	68	trazodone 100 mg	11	1
5	72	trazodone 150 mg	4	1

### Feasibility/Process Results

**Table 3** summarizes results related to the feasibility of the intervention (in preparation for a future randomized trial). Patients were willing to participate in the study. The main challenge to recruitment was financial—namely, the co-payment associated with 6 weekly sessions. Providing information on recruitment feasibility, one prospective patient who declined participation voiced “attachment” to his/her zolpidem prescription. Participants were very satisfied with the protocol, although they wished they could have received all study medications at the initial session. The participants’ health insurances covered a maximum of 30 days per prescription, which limited the supply that could be dispensed at the initial study visit. No participant disclosed using “extra” doses of hypnotics that were not part of their blister packs. No issues were encountered with preparation of the study medications. Participants’ satisfaction at post-treatment with the method of medication reduction was 0.4, indicating strong satisfaction (where 0 is strong satisfaction). Participants’ willingness to follow the same treatment again was 0.4 (where 0 is strong willingness). Finally, the participants’ assessment at post-treatment that they would follow the treatment for getting better sleep was 0.4, which indicates strong agreement.

**Table 3:** Feasibility results (N = 5 participants).

Recruitment barriers:	Main barrier was financial (i.e., co-payment associated with 6 CBTI sessions). One prospective participant was unwilling to taper her hypnotic (zolpidem).
Participant satisfaction with protocol:	Participants were very satisfied with the protocol, although they wished they could have had all study medications provided at the initial session instead of in two parts (4 weeks, 2 weeks), which was due to insurance covering 30 days maximum per refill.
Program credibility measures:	Baseline mean was 20 (range 18 to 21) out of a possible range 4 to 24, where 24 = most credible. Participants all rated credibility as 24 at post-treatment (credibility data was missing in 2 participants).
Confidence that participant would follow the program at baseline:	9 (range 6 to 10; possible range was 1 to 10, 10 = very confident)
Perceptions of importance of following the treatment to get better sleep:	9 (range 8 to 10; possible range was 1 to 10, 10 = very important).
Satisfaction at post-treatment with the method of medication reduction:	0.4 (range 0 to 1) out of possible range 0 to 4, where 0 = strongly agree
Willingness to follow the same treatment again:	0.4 (range 0 to 1) out of possible range 0 to 4, where 0 = strongly agree
Willingness to follow the treatment recommendations:	0.4 (range 0 to 1) out of possible range 0 to 4, where 0 = strongly agree

CBTI, cognitive behavioural therapy for insomnia.

### Medication and Sleep Results

All 5 participants had complete cessation of hypnotic use at post-treatment compared with baseline (5/5 were using hypnotic at baseline versus 0/5 at post-treatment). **Table 4** summarizes the changes in patient-reported sleep and mood measures. For the DBAS-16, 4/5 participants at baseline had a mean DBAS item score of ≥4 (threshold for unrealistic expectations about sleep) compared with 0/5 at post-treatment. For the Insomnia Severity Index, 4/5 of participants had “clinical insomnia” (ISI > 14) at baseline compared with 0/5 at post-treatment. For the Epworth Sleepiness Scale, 1/5 of participants had hypersomnolence (ESS > 10) at baseline compared with 0/5 post-treatment. For the Patient Health Questionnaire-9 (PHQ-9), 2/5 participants had at least mild depressive symptoms (≥5) at baseline compared with 0/5 at post-treatment. Two participants were assessed for benzodiazepine withdrawal (the other 3 were not taking benzodiazepine receptor agonists) and neither had concerning CIWA scores (both were in the low end of the “mild: 1–20” category).

**Table 4:** Sleep and mood measures descriptive statistics.

Measure	Mean (SD) at baseline	Mean (SD) at post-treatment	Difference between post-treatment and baseline means
Dysfunctional Beliefs and Attitudes about Sleep-16 item	4.8 (2.3)	1.1 (−0.8)	−3.7
Insomnia Severity Index	17.8 (3.3)	1.6 (2.1)	−16.2
Epworth Sleepiness Scale	4.6 (5.1)	2.0 (1.4)	−2.6
Patient Health Questionnaire-9	5.2 (7.6)	0.6 (0.9)	−4.6

SD, standard deviation.

**Figure 1:**
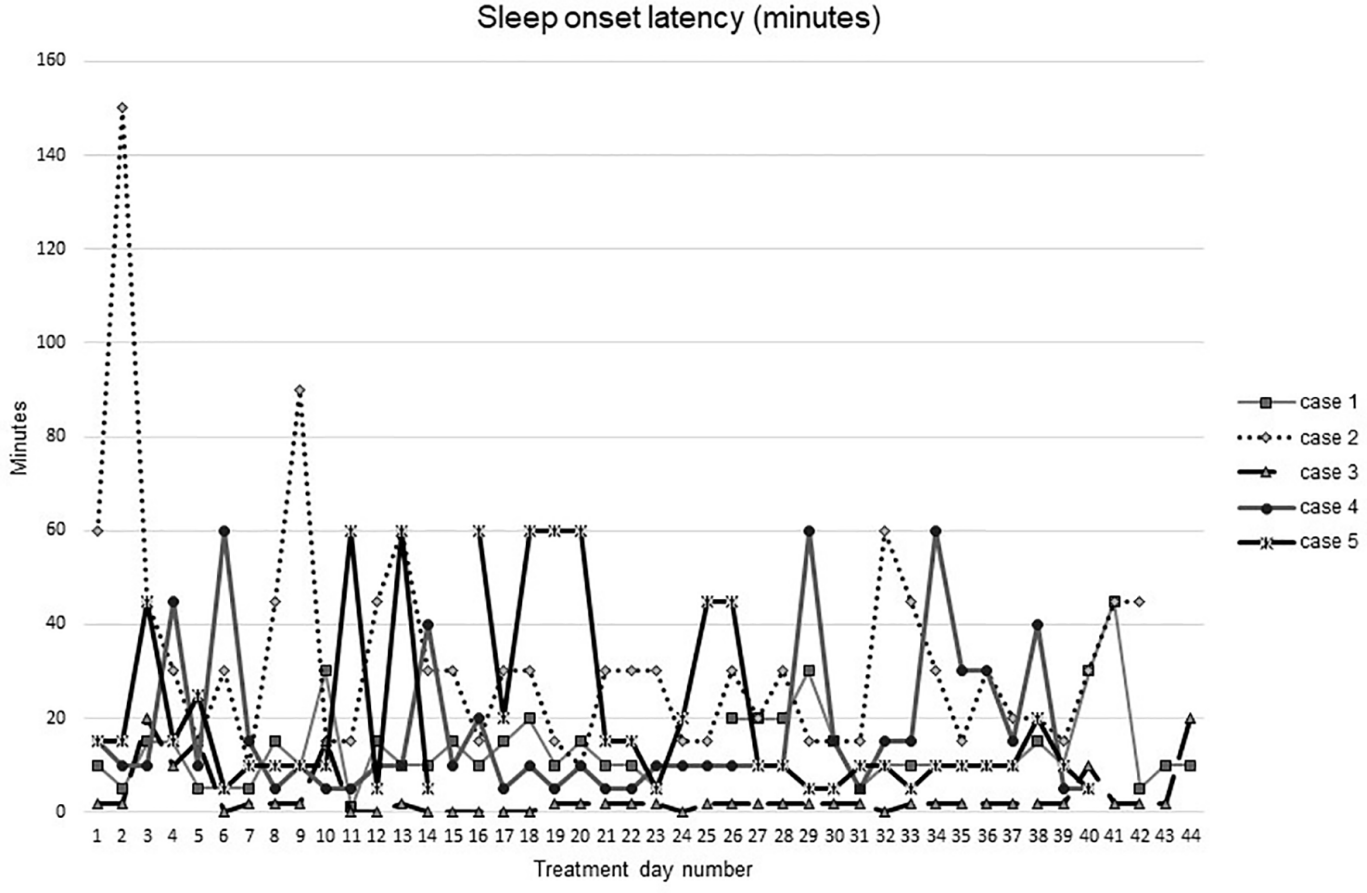
Sleep onset latency during intervention (cognitive behavioral therapy for insomnia and hypnotic taper).

**Figure 2:**
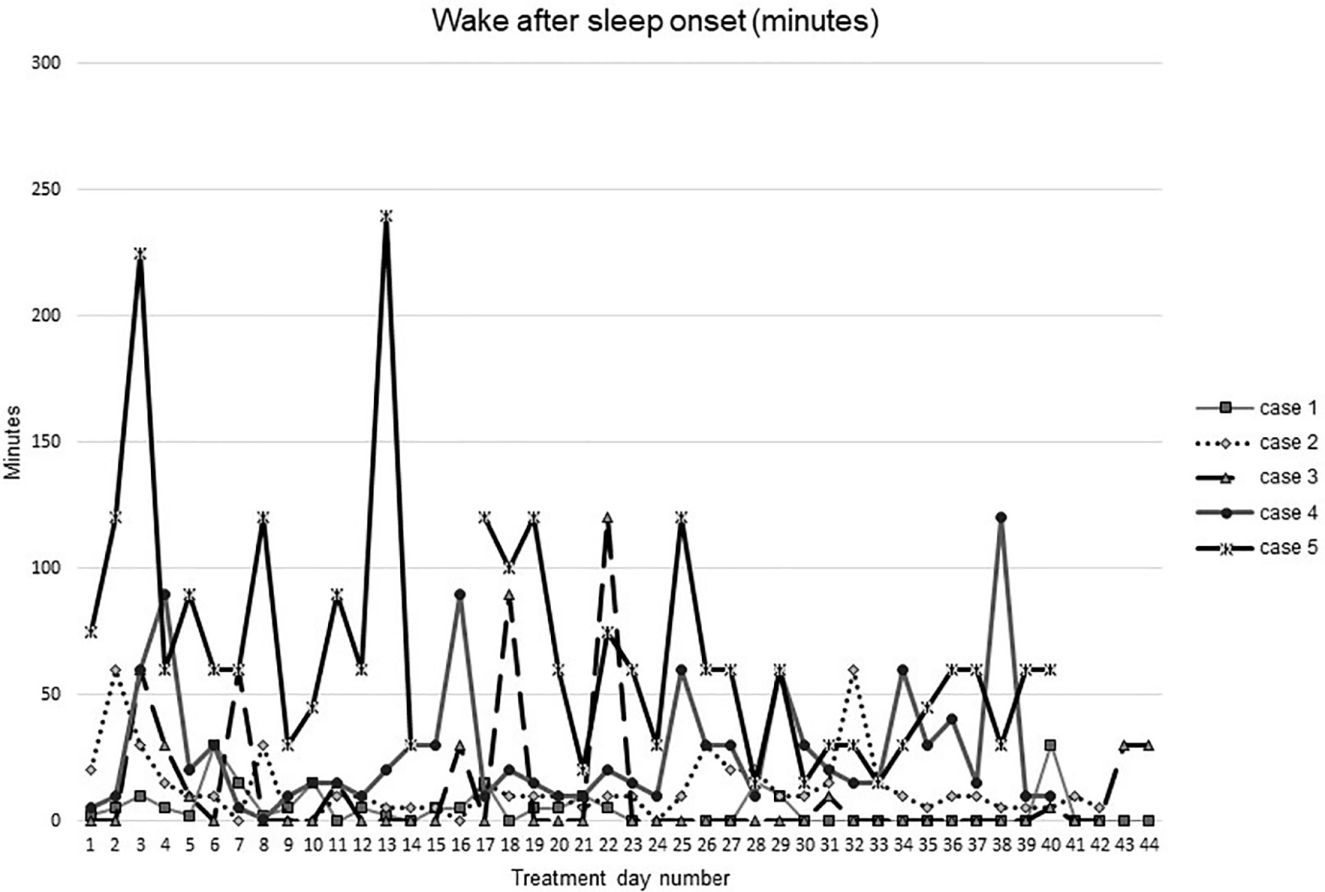
Wake after sleep onset during intervention (cognitive behavioral therapy for insomnia and hypnotic taper).

**Figure 3:**
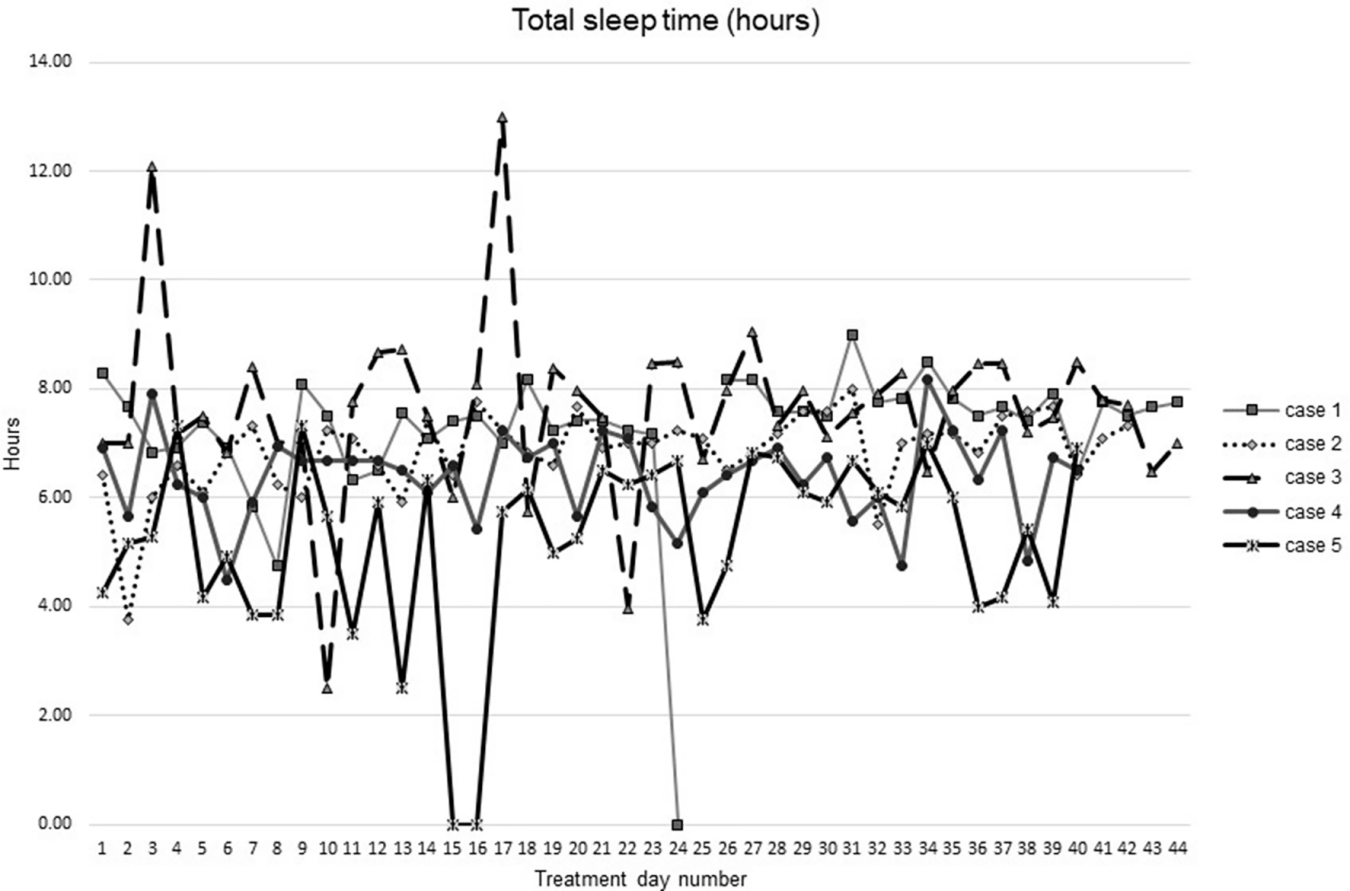
Total sleep time from during intervention (cognitive behavioral theraphy for insomia and hypnotic taper).

**Figure 4:**
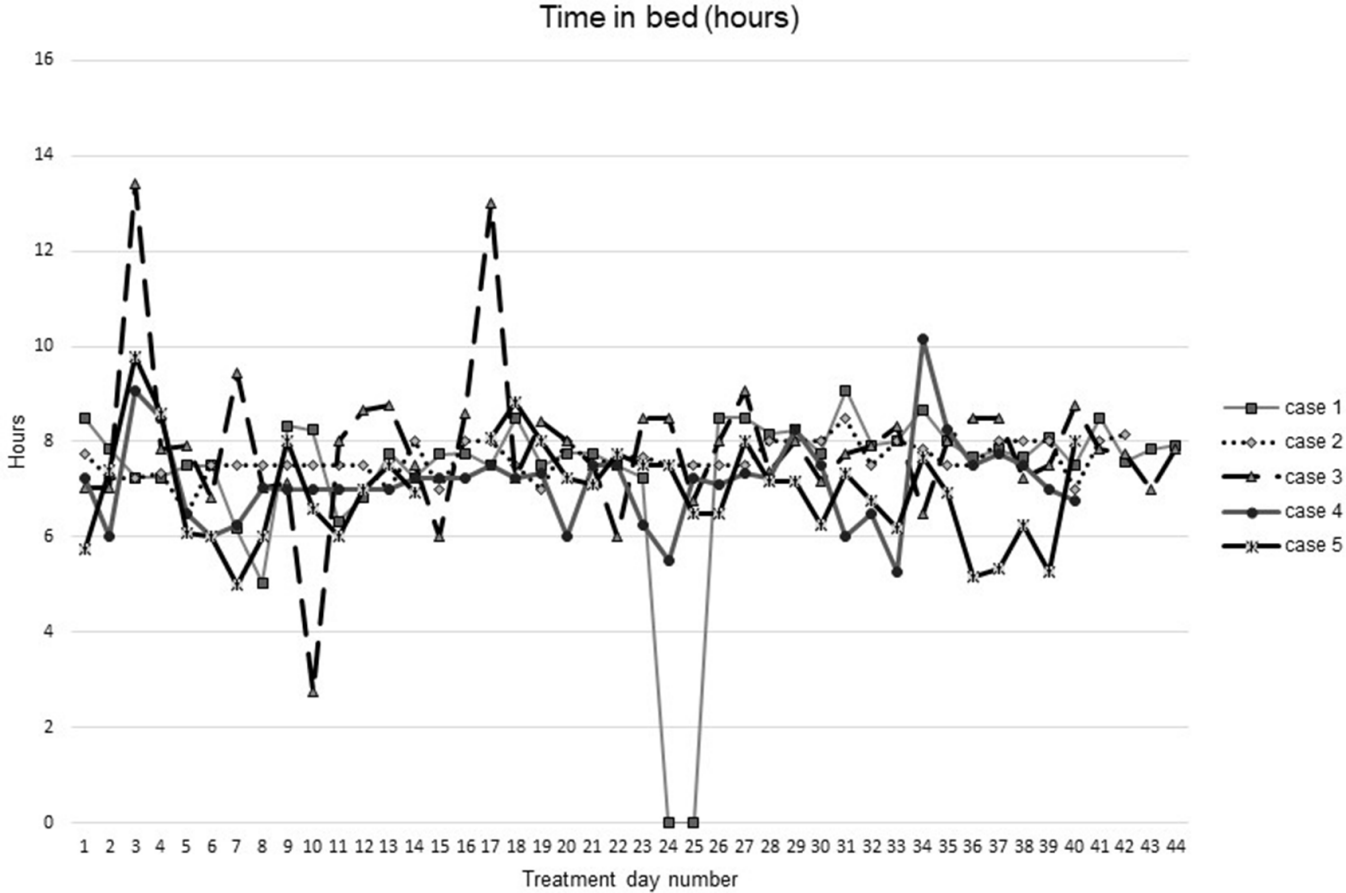
Time in bed during intervention (cognitive behavioral therapy for insomnia and hypnotic taper).

**Figure 5:**
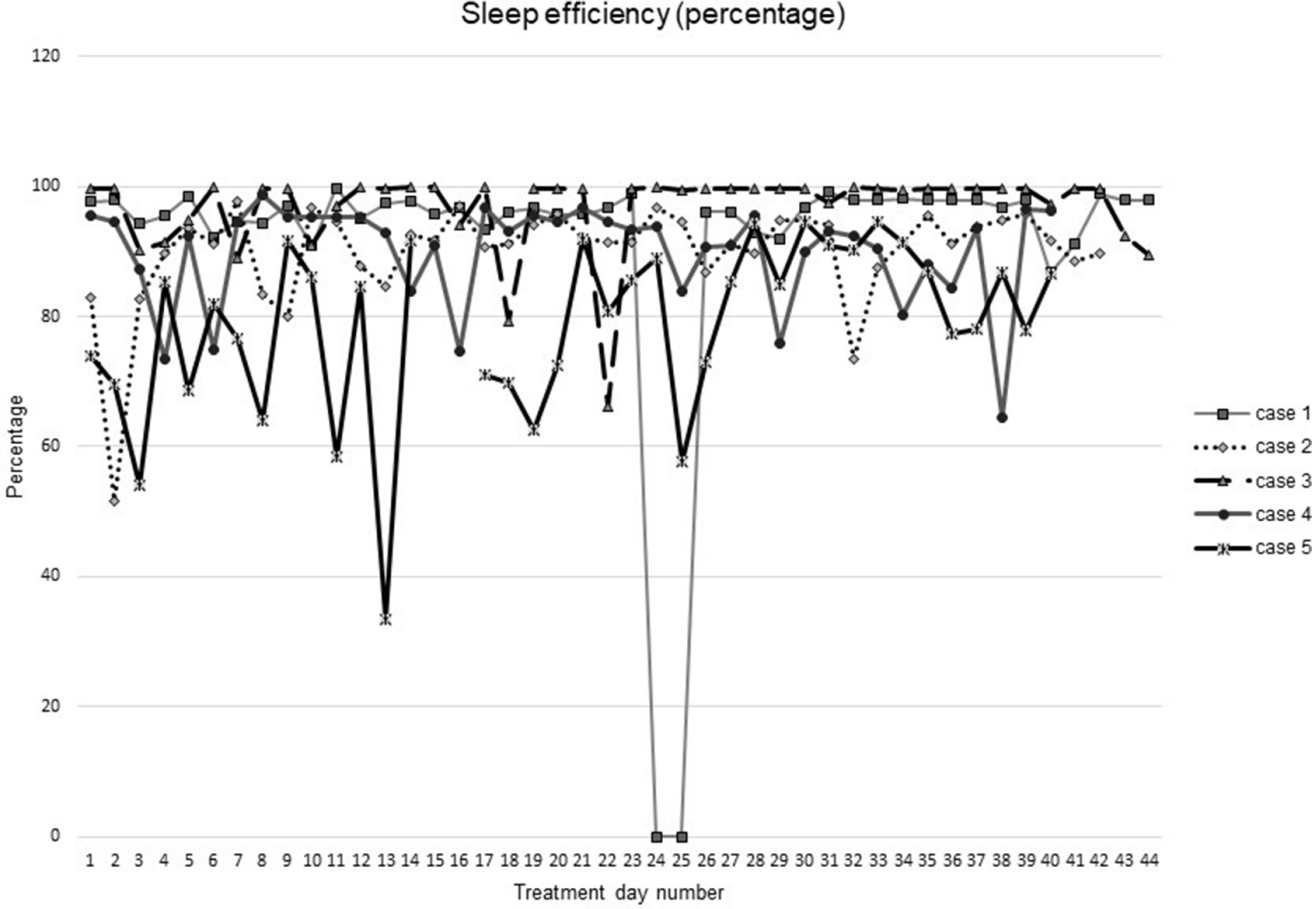
Sleep efficiency during intervention (cognitive behavioral therapy for insomnia and hypnotic taper).

**Figure 6:**
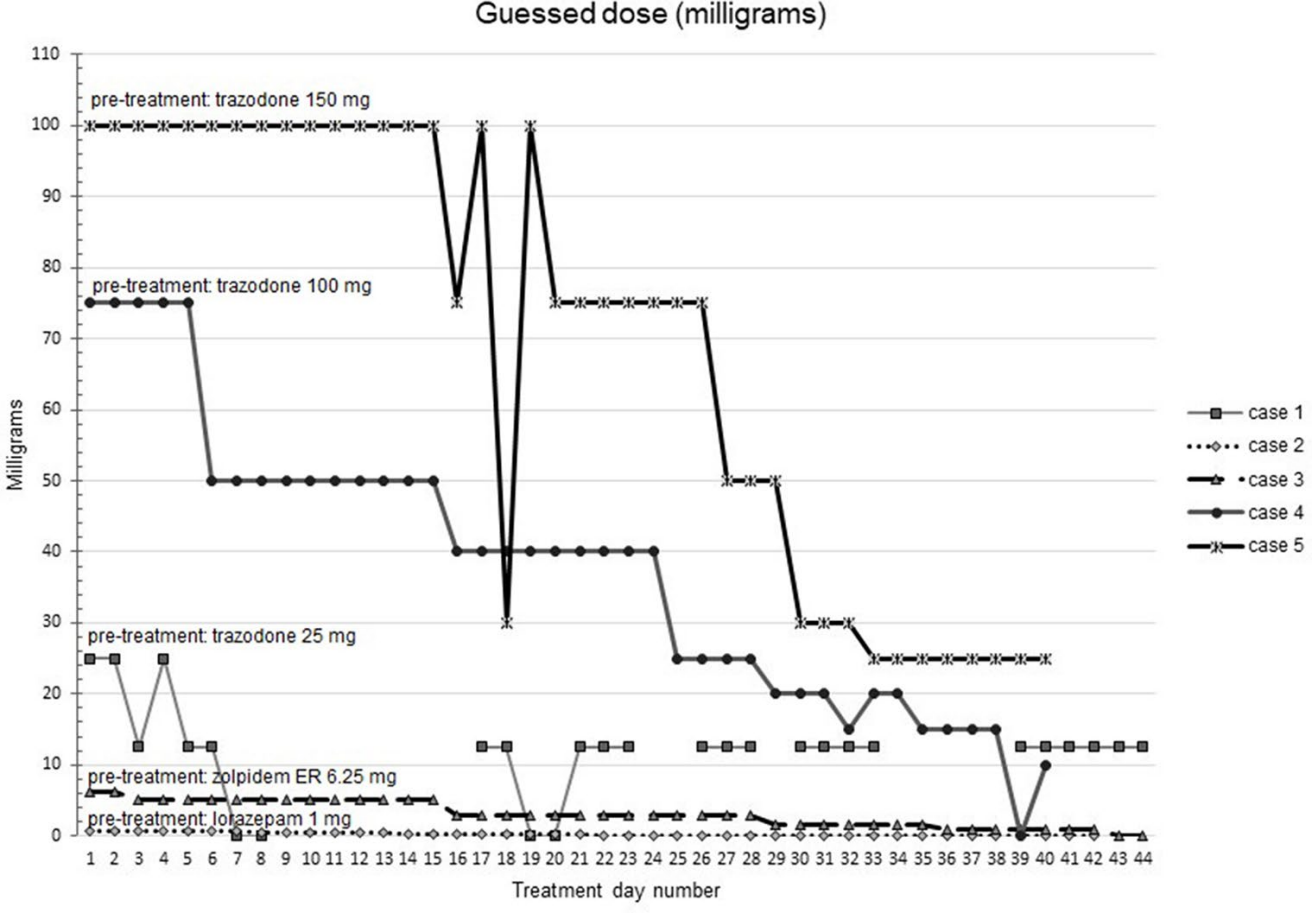
Participants’ beliefs about their hypnotic doses during intervention (cognitive behavioral therapy for insomnia and hypnotic taper). Participant 1 (baseline trazodone 25 mg) did not report any sleep on one day 24 during treatment due to an acute medical illness. Participant 2 took lorazepam 1 mg at baseline. Participant 3 took zolpidem extended release 6.25 mg at baseline. Participant 4 took trazodone 100 mg at baseline. Participant 5 took trazodone 150 mg at baseline.

**Figures 1**–**5** show diary-reported sleep onset latency, wake after sleep onset, total sleep time, time in bed, and sleep efficiency during CBTI and blinded hypnotic tapering. Overall, sleep efficiency improved from the treatment day 1 to the end of treatment. Participants’ sleep onset latency and wake after sleep onset remained the same or improved during treatment. **Figure 1** also shows the nightly guessed dose of medication for each participant. **Figure 6** shows the participants’ estimates of their nightly doses.

## Discussion

These results indicate that the addition of blinded tapering to a CBTI plus hypnotic taper protocol is feasible and may be beneficial to patients who are interested in discontinuing their hypnotic medication. The program was well-received by study participants. All participants were successfully drug-free at the end of the program. Insomnia symptoms, dysfunctional beliefs about sleep, and depressive symptoms improved. No patients experienced clinical withdrawal symptoms.

We believe the blinded taper protocol may improve hypnotic discontinuation success by reducing patient’s beliefs that they will experience withdrawal symptoms during tapering, such as increased insomnia or anxiety. These beliefs, also sometimes called expectancies, may have been formed during prior attempts to taper medications (i.e., direct experience), but also may develop through observing or speaking with friends or family who have attempted hypnotic discontinuation (21). Negative expectancies that occur when patients have been provided information about a drug that has been administered have been demonstrated in many studies. For example, a study of adult smokers found that participants reported greater reduction in nicotine cravings when informed that the nicotine-containing inhaler contained nicotine compared with when they were informed that the inhaler was nicotine-free, irrespective of the actual nicotine content (22). In a recent study of caffeine tapering, individuals assigned to a blinded tapering protocol reported fewer withdrawal symptoms than individuals assigned to open tapering where individuals were told their daily caffeine dose (8).

This feasibility study identified several issues that should be addressed in planning for a larger study. Participants in this study were responsible for paying costs associated with their study medications. A future study should provide study medication at reduced or no cost to participants. We also identified several issues that would help with data analysis of a future study including biomarkers of medication use. Finally, a longer follow-up time period would help with comparisons to published studies of duration of effects of the intervention.

Blinded tapering of hypnotic medications is a promising component that could be added to existing strategies. We are currently conducting larger, controlled trials [CBTI plus blinded taper versus CBTI plus standard (open) taper; NCT 03687086] testing the addition of blinded tapering to currently recommended approaches.

## Data Availability Statement

The datasets generated for this study are available on request to the corresponding author.

## Ethics Statement

The studies involving human participants were reviewed and approved by Providence Health and Services Institutional Review Board. The patients/participants provided their written informed consent to participate in this study.

## Author Contributions

CF, JM, CA, and LK contributed to the design of the study. LK contributed to data collection. CF and LK contributed to data analysis. CF, JM, CA, JD, IC, AM, AG, MZ, and LK contributed to interpretation of data and preparation of the manuscript.

## Funding

Research reported in this publication was supported by the Department of Veterans Affairs [IIR 17-234], UCLA Department of Medicine, National Institute On Aging of the National Institutes of Health [K23AG045937, K23AG049955, and 1R01AG057929-01], The Beeson Career Development in Aging Research Award Program (supported by NIA, AFAR, The John A. Hartford Foundation, and The Atlantic Philanthropies), and National Institute of Heart, Lung and Blood [K24 HL143055]. The content is solely the responsibility of the authors and does not necessarily represent the official views of the National Institutes of Health, UCLA, or the Department of Veterans Affairs.

## Conflict of Interest

The authors declare that the research was conducted in the absence of any commercial or financial relationships that could be construed as a potential conflict of interest.
